# Superomedial pedicle skin-reducing mastectomy in ptotic and large-sized breasts with two-stage reconstruction through transaxillary video-assisted technique: An effective surgical and anesthetic approach

**DOI:** 10.3389/fsurg.2022.1040602

**Published:** 2023-01-09

**Authors:** G. Di Monta, U. Marone, F. Avino, E. Esposito, V. Cepparulo, E. Morra, R. Saponara, F. Bifulco, A. Cuomo, M. Cascella, S. Mori

**Affiliations:** ^1^ UOSD Oncoplastic Surgery, Istituto Nazionale Tumori IRCCS Fondazione “G. Pascale”, Naples, Italy; ^2^ UOC Breast Surgery, Istituto Nazionale Tumori IRCCS Fondazione “G. Pascale”, Naples, Italy; ^3^ UOC Division of Anesthesia, Istituto Nazionale Tumori IRCCS Fondazione “G. Pascale”, Naples, Italy

**Keywords:** conservative mastectomies, breast reducing surgery, reduction mammaplasty, endoscopic breast reconstruction, Erector Spinae block, Serratus Anterior Plane block, opioid-free anesthesia

## Abstract

**Introduction:**

Skin-reducing mastectomy has been applied to several surgical techniques in which subcutaneous mastectomy is associated with various types of skin reduction, with preservation of a lower dermal flap to reinforce the inferior lateral seat of an implant. The aim of the study is to present a case series of patients with pendulous/ptotic and/or large-sized breasts treated for breast cancer at the Breast Surgery Unit of Istituto Nazionale Tumori IRCCS Fondazione “G. Pascale”, Naples, Italy, with the superomedial pedicle skin-reducing mastectomy technique, two-stage reconstruction, and transaxillary video-assisted technique, when a postoperative radiotherapy was indicated. We verified its effectiveness by discussing its results, especially in patients who are candidates for postmastectomy radiotherapy.

**Materials and methods:**

A single-center retrospective study was performed between January 2020 and March 2021 on a prospectively filled database of conservative mastectomies. Of the 64 patients who underwent nipple/skin-sparing mastectomies in the mentioned period, 17 (mean age 46 years, range 30–62 years) were treated with superomedial pedicle skin-reducing mastectomy, with two-stage breast reconstruction through transaxillary video-assisted replacement expander with definitive prosthesis and contralateral symmetrization, selected for postmastectomy radiotherapy.

**Results:**

We had only three minor complications. No flap necrosis, no infections, no breast seromas, and no reconstructive failures were observed. During follow-up of the patients treated with video-assisted reconstruction, there were no cases of infection, hematoma, implant rupture, or suture dehiscence in the reconstructed breast.

**Discussion:**

Skin-reducing mastectomy with superomedial pedicle is a safe and reliable procedure to treat breast cancer in selected patients, i.e., those with pendulous/ptotic and or large-sized breasts. Particularly, in patients who undergo postmastectomy radiotherapy, the two-stage reconstruction with video-assisted transaxillary endoscopic approach can find its main indication, using incisions positioned far from the mammary region, offering numerous advantages.

## Introduction

Conservative mastectomies are currently validated as safe and reliable surgical procedures from an oncologic point of view. The size and location of the tumor, the size of the breast, the potential neoadjuvant and adjuvant treatments are the main factors influencing the surgical choice. Small breasts are reconstructed using permanent prosthetic implants or temporary expanders, using surgical access with S italic or periareolar incisions. Medium and large breasts are usually accompanied by varying degrees of ptosis and, therefore, require different skin-reduction procedures, with a prevalent vertical pattern or an inverted T pattern with contralateral symmetrization, to obtain a more adequate cosmetic result (1).

For patients anatomically characterized by various degrees of ptosis and breast hypertrophy, with the need for repositioning of the nipple–areola complex (NAC) and skin reduction during the surgical procedure of mastectomy, in the early 1990s, Bostwick proposed a surgical technique with packaging of a combined musculocutaneous pocket able to accommodate permanent implants in order to obtain the best possible esthetic result (2).

Hammond et al. then revisited this technique, combining Carlson's type IV conservative mastectomy with simultaneous reduction of the breast skin and the preparation of a lower dermal flap to create a dermomuscular pocket to accommodate the implant, to give better coverage and reinforce its lower pole, reducing its potential exposure in the event of a skin incision break down, especially at level of the triple point of the inverted “T” scar (3, 4). Subsequently in 2006, Nava et al. introduced the term skin-reducing subcutaneous mastectomy, presenting a technique substantially similar to that described by Hammond et al., associating a mastectomy with skin reduction of type IV sec. Carlson to the packaging of a dermomuscular pocket that would allow the lower pole to adequately accommodate a definitive prosthesis (5). The technique of Nava et al. has been studied and programmed for patients with large breasts and various degrees of ptosis, both therapeutic and prophylactic in BRCA-mutated patients.

Currently, the term skin-reducing mastectomy has been applied to several surgical techniques in which subcutaneous mastectomy is associated with various types of skin reduction, with preservation of the lower dermal flap to reinforce the inferior lateral seat of an implant (1).

The purpose of this study is to present a series of patients with pendulous/ptotic and/or large breasts treated for breast cancer at the Breast Surgery Unit of the “Istituto Nazionale Tumori IRCCS Fondazione G. Pascale,” Naples, Italy, with the technique of the upper mastectomy to reduce the skin of the medial pedicle, reconstruction in two stages, and replacement of the video axillary expander with a permanent implant and contralateral symmetrization, in cases where postoperative radiotherapy is indicated, verifying its effectiveness and discussing the results, above all in patients who are candidates for postmastectomy radiotherapy (PMRT).

## Materials and methods

### Patient selection

A single-center retrospective study was performed between January 2020 and March 2021 on the conservative mastectomies database of Istituto Nazionale Tumori IRCCS Fondazione “G. Pascale,” Naples, Italy. In the mentioned period, 64 patients underwent nipple/skin-sparing mastectomies, and 17 (mean age 46 years, range 30–62 years) were treated with superomedial pedicle skin-reducing mastectomy, with two-stage breast reconstruction through transaxillary video-assisted replacement expander with definitive prosthesis and contralateral symmetrization. In those patients, preoperative multidisciplinary evaluation had indicated the need for PMRT (Table 1). A team of surgical oncologists work together with the oncoplastic surgery team. Inclusion criteria were breast cancer (Tis-T3) with multifocal or multicentric disease in women with pendulous/ptotic and large-sized breasts (nipple–sternal notch distance longer than 25 cm and an areola–inframammary fold distance longer than 8 cm), with no evidence of skin involvement, unsuitable for breast conservation and after neoadjuvant chemotherapy at which time conservation was still not indicated. The study was conducted in accordance with the Declaration of Helsinki (as revised in 2013). Ethical approval was not required by the Ethics Committee since this is a retrospective case series analyses according to SAGE guidelines. All patients gave written informed consent before having their surgery.

**Table 1:** Main characteristics of patients submitted to superomedial pedicle skin-reducing mastectomy.

Age (years)	Tumor type	SLNB	AxD	Neoadjuvant therapy	Implant Volume	Type of implant	PMRT
37	T1 + DCIS	No	Yes	No	275	Expander	Yes
62	LABC Bil.	Yes	Yes	Yes	550	Expander	Yes
30	LABC	No	Yes	Yes	350	Expander	No
51	LABC	No	Yes	Yes	450	Expander	No
41	LABC	No	Yes	Yes	350	Expander	Yes
43	LABC	No	Yes	Yes	350	Expander	Yes
51	LABC	No	Yes	Yes	550	Expander	Yes
54	T2	Yes	No	No	440	Prosthesis	No
57	T1 Multic.	Yes	No	No	300	Prosthesis	No
39	T2 + DCIS	No	Yes	No	350	Expander	Yes
36	LABC	Yes	No	Yes	450	Expander	Yes
42	LABC	Yes	No	Yes	245	Prosthesis	No
46	T1 Multic.	Yes	No	No	450	Expander	No
56	T2N1	No	Yes	No	550	Expander	Yes
53	T2N1	No	Yes	No	450	Expander	Yes
56	T1 + DCIS	Yes	No	No	450	Expander	No
37	T1 + DCIS	Yes	No	No	350	Expander	No

LABC, locally advanced breast carcinoma; DCIS, ductal carcinoma in situ; SLNB, sentinel lymph node biopsy; AxD, axillary dissection; PMRT, postmastectomy radiotherapy; Multic., multicentric; Bil., bilateral.

Heavy smokers and patients with secondary microvascular disease (diabetes, postradiation therapy, etc.) were excluded. In all patients, the neoplastic nipple–areola complex involvement may be predicted before surgery and assessed intraoperatively. The choice between definitive prosthesis and temporary expander was above all conditioned by the indication to postoperative radiotherapy and anatomical characteristics on the breasts especially in relation to the tissues quality and thickness. Postoperative complications such as mastectomy flap necrosis, wound dehiscence, infection, breast seroma, hematoma, and areolar necrosis were recorded.

### Surgery technique

**Figure 1:**
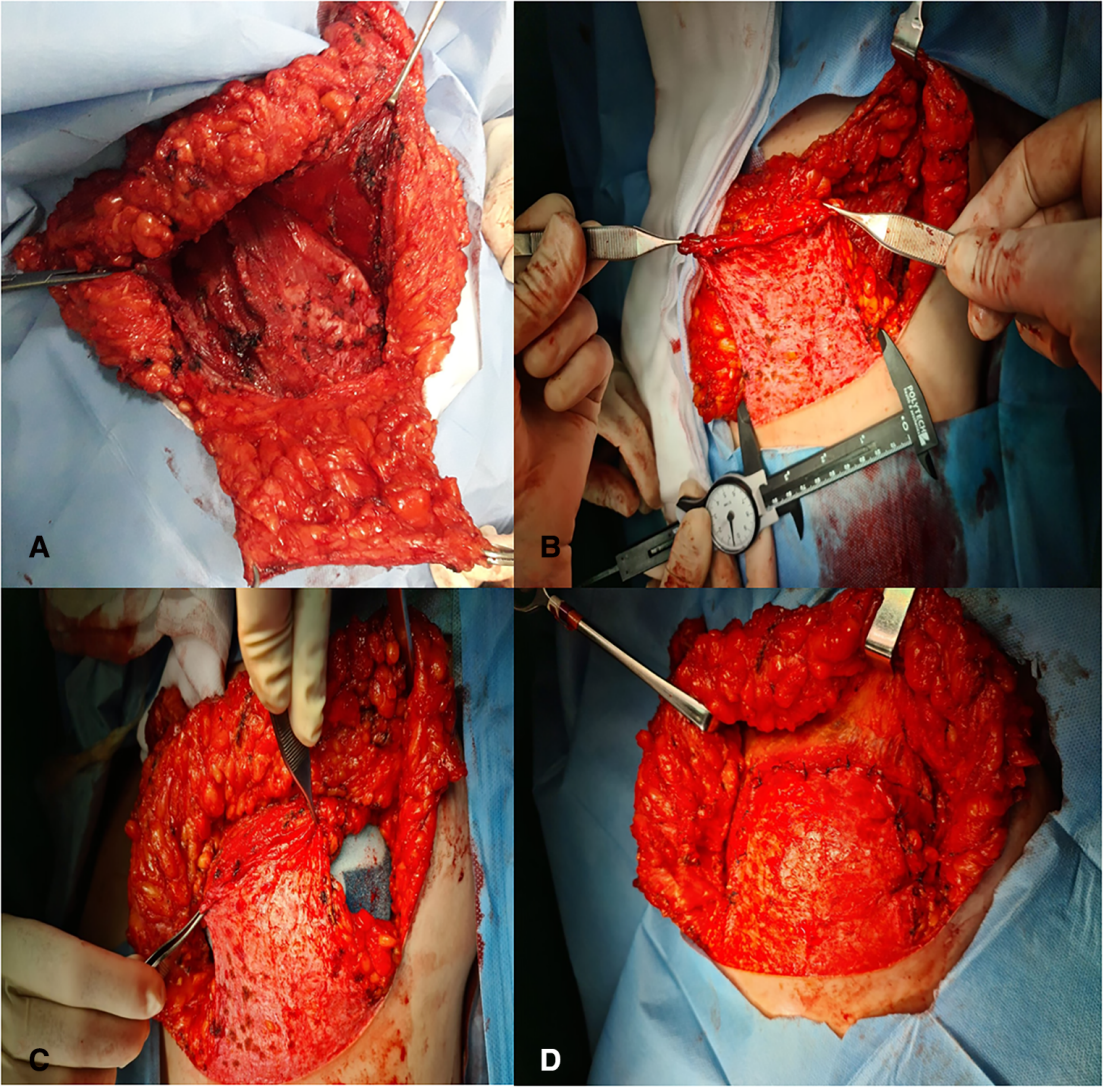
(**A,B**) Subpectoral pouch closed inferiorly and laterally by the dermal flap with imitated lift of the serratus anterior. (**C,D**) Tissue expander or definitive prosthesis placed in the dermomuscular pocket.

Patients were placed in an upright position, and the mark ups for the Wise keyhole skin pattern reduction are performed. The superomedial pedicle is marked from the center of the new areola position, passing around the NAC to the point of the medial limb of the keyhole. According to the inverted T-technique, skin reduction is carried out with accurate de-epithelialization, and a lower dermal flap was set up to be subsequently joined to the incised pectoralis major muscle. The blood supply of the nipple is secured from superomedial pedicle dermal flap. Henceforth, the removal of the entire mammary gland is performed according to the inverted “T” incision. It is important to note that during the removal of the entire glandular tissue and epipectoral preparation, sparing the II-IV perforator vessels is of high priority as these provide blood supply to the medial skin flap and thereby for the nipple. After the whole breast has been removed, a subpectoral pouch is created and closed inferiorly and laterally by the dermal flap with imitated lift of the serratus anterior. Then, the tissue expander or definitive prosthesis should be placed in the dermomuscular pocket (Figures 1A–D). The NAC is then rotated 90° upward (the 9-o'-clock point on the areola now becomes the 12-o'-clock or superior most point of the areola) and laterally inset into its new position. The triple point is then closed, and this is followed by closure of the inferior limbs of the keyhole, thus reestablishing the new breast shape. Then, an axillary dissection or sentinel lymph node biopsy was done *via* a separate incision.

**Figure 2:**
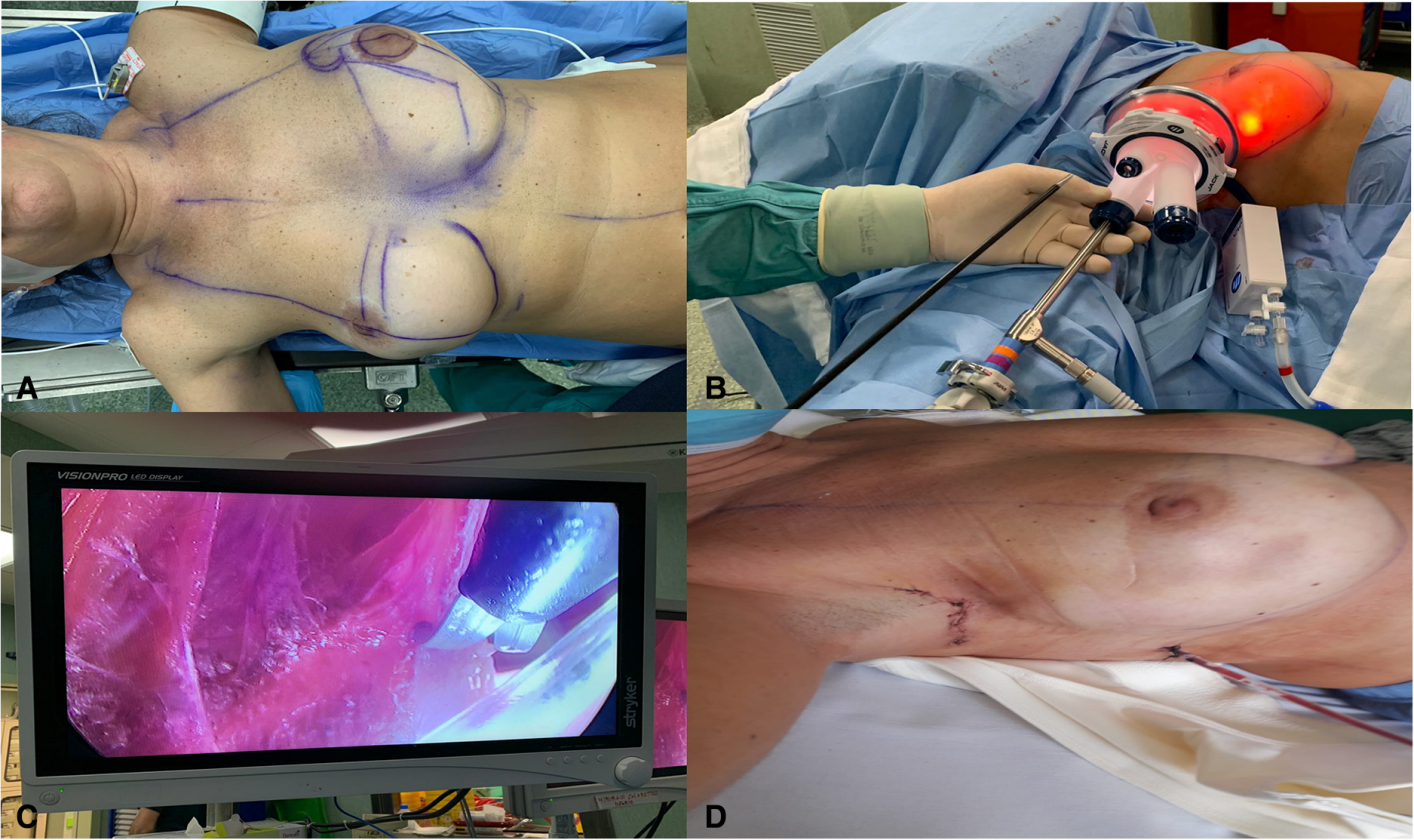
(**A**) Patient positioned supine on the operating table with the arms extended and held in place of 90° at the height of the axilla. (**B**) Axillary incision and introduction of a single endoscopic port for endosurgery. (**C**) Endoscopic-guided circumferential capsulotomy. (**D**) Immediate postoperative view.

In our series, in the patients treated with postmastectomy radiotherapy, we conducted reconstruction in two stages with video-assisted technique through the axillary incision performed for sentinel lymph node biopsy or lymphadenectomy. Patients were positioned supine on the operating table with arms extended and held in a 90° position at the axilla (Figure 2A). The same axillary incision previously used for sentinel node biopsy or axillary dissection is revisited, and a superficial subcutaneous dissection is performed to reach the lateral border of the pectoralis major muscle, through minimal access by inserting a single endoscopic port for endosurgery (Figure 2B). Under direct visualization with an endoscopic camera and an endo-scalpel, the pectoralis major muscle was carefully detached and rigorous hemostasis was performed with access to the submuscular pocket. Subsequently, it was possible to remove the expander, open and dissect the scarred area with capsulectomy, where possible, and endoscopically guided circumferential capsulotomy, in order to revise the new pocket, and model the new inframammary sulcus as previously marked and avoid overexpansion of the upper post (Figure 2C). Once the submuscular pocket was completed, we inserted the definitive prosthesis while monitoring the condition of the skin, and then inserting a drain and closing the brief axillary incision (Figure 2D). Simultaneously, symmetrization with the contralateral breast was performed to obtain the highest degree of symmetry possible, after inserting a bilateral drain and closing the axillary incision with subcutaneous and intradermic absorbable sutures.

### Anesthesia technique

The anesthetic approach involves the association of two anesthetic blocks with general anesthesia. The anesthetic blocks are the Erector Spinae Plane (ESP) block and the Serratus Anterior Plane block. The former is implemented at the T4 level with ultrasound guidance by using ropivacaine 0.5%, mono- or bilaterally on a case-by-case basis (e.g., plastic surgery on the contralateral breast). The ultrasound-guided Serratus Anterior Plane Block is performed (ropivacaine 0.5%) at the fourth rib. In both blocks, local anesthetic volumes are adjusted according to the maximum safe dose.

After the blocks, the patient is transferred to the operating room, where an opioid-free general anesthesia strategy is employed. In particular, the protocol includes propofol (2 mg/kg), ketamine (1 mg/kg), rocuronium bromide 0.6 (mg/kg), orotracheal intubation, and maintenance of anesthesia with sevoflurane (MAC at 0.8). Postoperative prophylaxis of nausea and vomiting is administered according to the APFEL score. At the end of the operation, paracetamol (1 g) is administered i.v. and sugammadex is used to reverse neuromuscular blockade, based on the train of four (TOF) value.

## Results

**Figure 3:**
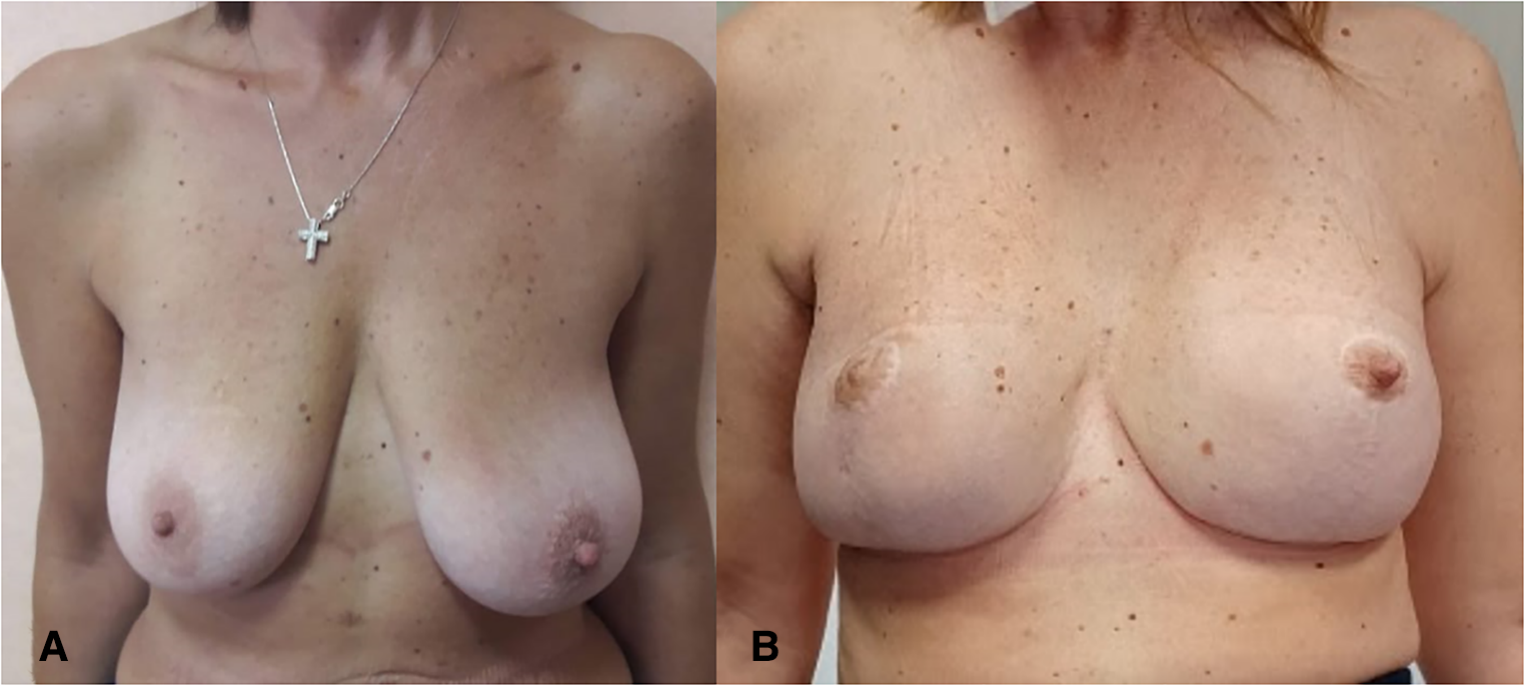
(**A**) Preoperative view of a patient affected by right side breast cancer and BRCA mutation. (**B**) Nine-month postoperative view.

**Figure 4:**
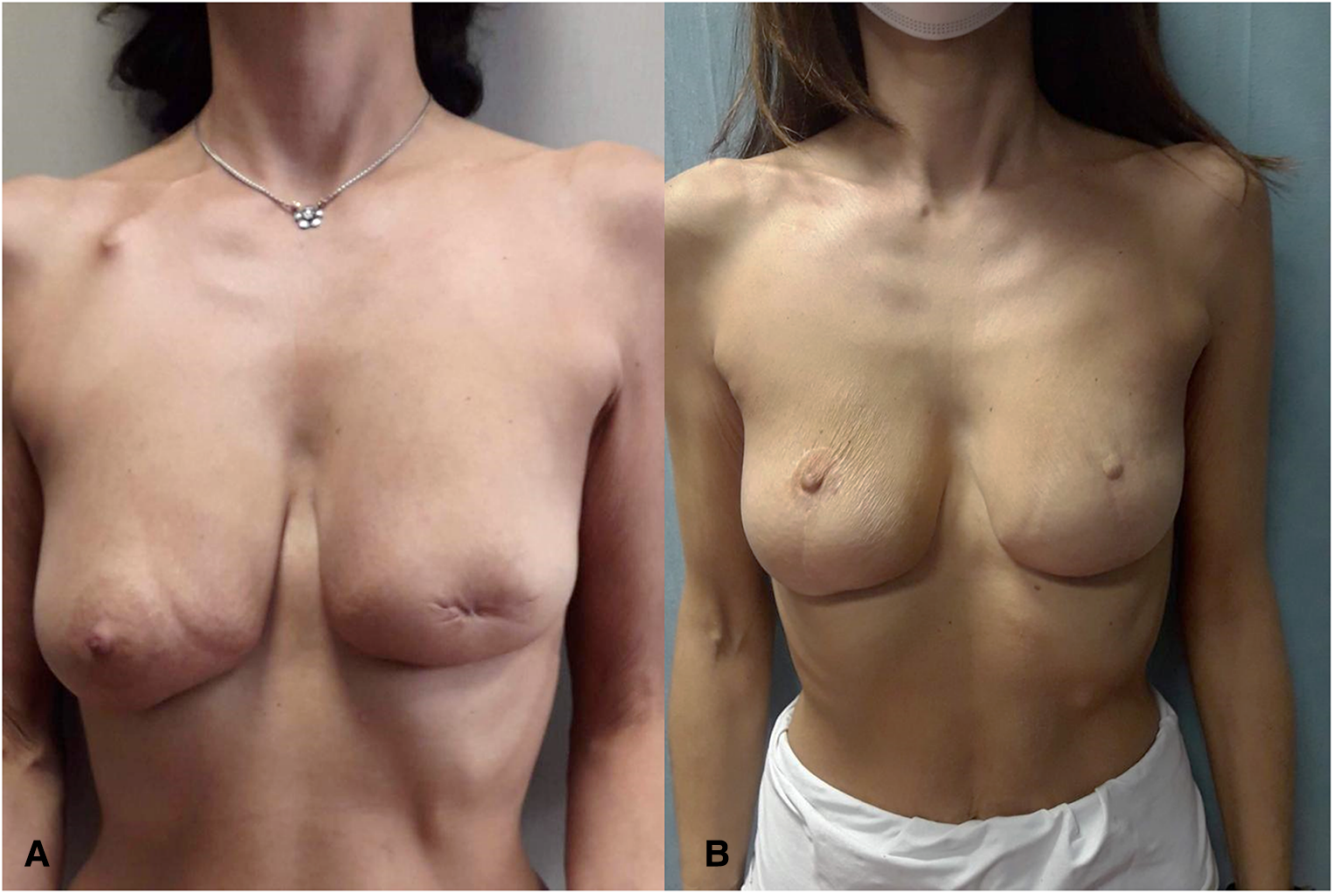
(**A**) Preoperative view of patient treated in another hospital with left-side central quadrantectomy with resection of the BRCA-mutated nipple–areola complex. (**B**) Twelve-month postoperative view.

Of the 17 superomedial pedicle skin-reducing mastectomies, all were curative: in two cases, the nipple–areola complex was removed because tumor cells were involved. The median follow-up was 10 months (range 5–19 months). Eight patients (47%) were treated surgically after neoadjuvant chemotherapy. In all patients at risk of postoperative radiotherapy, reconstruction was performed primarily with an expander (medium size implant was 400 ml, the largest 550, and the smallest 245 ml), and after some months at the end of radiotherapy treatment, they were submitted to the second reconstructive stage procedure with transaxillary video-assisted expander substitution with permanent implant and contralateral symmetrization (Figures 3, 4). Only patients in whom the preoperative evaluation had not indicated a possible postsurgical radiotherapy and for the quality of the cutaneous and subcutaneous tissues, reconstruction was performed with immediate prosthesis together with symmetrical procedure on the opposite site at the same time. We had only three minor complications: two patients developed partial NAC necrosis, which was resolved by conservative treatment. Only one patient developed area of cutaneous suffering around the junction of the “T” scar, which was later healed by secondary intention. No flap necrosis, no infections, no breast seromas, and no reconstructive failures were observed.

During follow-up of the patients treated with video-assisted reconstruction, there were no cases of infection, hematoma, implant rupture, or suture dehiscence in the reconstructed breast.

## Discussion

In patients suffering from breast cancer with voluminous and ptotic breast anatomical features, to obtain an adequate oncological root and a good cosmetic result, it is necessary to perform conservative mastectomy with a variable degree of skin reduction. Subcutaneous Wise pattern mastectomy was the one mostly used in this sense until a few years ago; however, it was burdened by a high complication rate (up to 27%) (3). In 2002, Hammond et al. described a new technique in which immediate postmastectomy breast reconstruction made use of an inferior dermal flap associated with inverted “T” skin reduction, in order to create a combined dermomuscular pocket to accommodate, in most cases of patients, a temporary expander, followed by its replacement with a permanent prosthetic implant at a later time (4). In their work, Hammond et al. emphasized the concept that the creation of this well-vascularized dermomuscular combination pocket provides a solid and stable cover for the implant, protecting it from exposure if the wound is opened. Furthermore, the preparation of the lower dermal flap allows the creation of a pocket for housing the implant larger than that which could be provided using only the muscles of the chest wall, reducing the tension on the underlying prosthetic device. Finally, Hammond et al. reaffirm the concept that the creation of the dermomuscular pocket allows to add more fluid to the expander, thus reducing the number of postoperative expansions, favoring the psychological benefit of the patient, already creating an adequate breast volume, in the immediate postoperative period. Subsequently, Nava et al. described their technique of conservative skin-reducing mastectomy in an expansive manner in technical steps, with immediate reconstruction and minimal incidence of complications (5). This last procedure has been particularly successful and followed by the best and most up-to-date surgical and reconstructive breast care teams, also undergoing improvements, as in the work of Colizzi et al., in which the lower dermal flap is used as described by Nava et al. but increasing its mobility through a partial detachment of the lateral portion of its insertion along the inframammary fold, allowing the preservation of the anterior dentate or its limited lifting, so as to adequately close the pocket inferiorly and laterally, reducing the risks of lateral displacement of the implant and improving the lateral contour of the breast, giving it a more natural shape (6).

In our patient series, we used skin-reducing mastectomy as described by Nava et al., using the superomedial pedicle as an interesting alternative approach in women with large and/or ptotic breasts. This is the same technique used in esthetics and was described for the first time in 1975 by Orlando and Guthrie as a method of transposition of the nipple–areola complex using a superomedial dermal pedicle in breast reduction. Several studies, which used this surgical approach, have demonstrated its safety with its use in breast reduction in patients with large breasts, with a complication rate equivalent to that using the lower pedicle reduction technique (7–12). Bauermeister et al. reported the most significant retrospective review of 938 breast reduction mammoplasties performed in a single institution over a 10-year period, using the superomedial pedicle breast reduction technique, without major complications, such as necrosis of the areola–nipple complex and/or skin flaps, with only minor complications favorably treated with conservative dressings (14). In our series of patients, we performed skin-reducing subcutaneous mastectomy, using the superomedial flap, as in esthetic mammoplasty, with the same preoperatory planning. The afternoon preceding the surgery, the patient was placed in a frontal and upright position, tracing the well-known landmarks, the sternal midline, the lines that draw the breast meridian, and the Pitanguy point to mark the new position of the nipple–areola complex. We then proceeded with the design of the Wise pattern, being very careful in designating the superomedial pedicle, with an adequately wide base, to allow an adequate vascular supply, without undermining it. During surgery, all the mammary tissue was removed in a mediolateral direction, taking extreme care not to damage the pedicle so as to avoid its devascularization, after having ascertained the absence of tumor involvement of the retroareolar tissue at the intraoperative histological examination. The pedicle, after having placed the implant in the dermomuscular housing pocket, was transposed into the position of the neo-areola.

The current trend is to perform breast reconstruction simultaneously with oncological surgery, with implant placements in the prepectoral or subpectoral area, in particular in relation to the quality of the patient's tissues and the possible indication for a PMRT. However, there are still a considerable number of mastectomized patients who undergo two-stage reconstruction, both due to the nature of their disease and the possible execution of PMRT, resulting in poor tissue quality and reduced skin healing capacity. This increases the risk of wound suffering when the same mastectomy scars are used as an access route to replace the expander with permanent implants, increasing the risk of extrusion and implant loss.

Vanni et al. (13) correlated surgical stress with a higher rate of postoperative complications. The surfaces of breast implants (textured or smooth) represent an immunological stimulus. Their prospective study (BIAL2.20) evaluated postoperative leukocyte response at baseline and postoperative day (POD) 1 and 2 after implant-based breast reconstruction. They concluded that textured implants had a statistically significant impairment in T-helper tendency during POD1 and POD2 compared to smooth implants by two-way ANOVA between groups.

In order to try to minimize tissue healing problems following PMRT and their potential negative effect on the expander or prosthesis, we performed a transaxillary endoscopic approach in these patients, replacing the temporary implant with the definitive one, thus avoiding retracing the previous mastectomy scar. Once accessed through the axillary scar of a previous lymphadenectomy or sentinel lymph node biopsy, it is then possible to remove the expander, carry out a video-assisted endoscopic remodeling of the dermomuscular pocket, and then place the definitive prosthesis, positioning the access scar outside the mammary gland, being certain that since the scar within a previously irradiated anatomical region is not in direct contact with the prosthesis in depth, any suffering will have no negative impact on the implant. This approach also allows, when necessary, the symmetrization of the contralateral breast, with a much clearer idea of the final volume of the reconstructed breast.

Serra-Mestre et al. described the first report of a new application of endoscopic surgery in delayed prosthetic breast reconstruction *via* the same incision created for axillary lymph node dissection or sentinel node biopsy, assessing the safety of the technique (14). In their study, 62 mastectomy patients were operated with inserting, as first step, a progressively filled expander, with endoscopic procedure thorough the axillary incision used for axillary surgery, placing the scar outside the breast, thus avoiding any tension with the filling of the expander and allowing the injection of the definitive volume in the expander in 100% of cases. After 3 months, the expander was replaced with a definitive prosthesis using the same incision after performing an endoscopy-guided circumferential capsulotomy. This technique has been used to avoid that the area subjected to maximum tension following the progressive filling of the expander and subsequently radio-treated was in direct contact with a scar reused to access the previous mastectomy, meaning that any problem that may occur during wound healing could also affect the implant. During follow-up, no cases of infection, hematoma, implant failure, or suture dehiscence in the reconstructed breast were reported. In all cases, it was possible to fill the expander with the definitive volume in the first surgical stage with high satisfaction in most of patients. They underline the concept that the indication to make delayed breast reconstruction increases in the setting of previous radiation, because of the poor tissue quality and healing capacity of the skin, increasing the risk of wound dehiscence when the same mastectomy scar is used as the access route. Using an access distant from the scar previously used in the mastectomy, therefore not re-entering through a scar present in a previously irradiated area, reduces scarring complications up to the extrusion and loss of the implant.

Super-medial pedicle skin-reduction mastectomy is a safe and reliable procedure for the treatment of breast cancer in selected patients, such as those with pendulous/ptotic and/or large breasts. The superomedial component of the pedicle incorporates adequate blood flow to the nipple–areola complex through the internal thoracic system, proving to be a valid anatomical, safe, and reliable choice in esthetic mammoplasty and also in the execution of conservative mastectomies.

The medial aspect of the pedicle retains the perforators from the internal thoracic vessels, whereas the superior component creates a wider pedicle, adding to its safety.

Selber (15) reported robotic nipple-sparing mastectomy (R-NSM) as the next step in the evolution of minimally invasive breast surgery. R-NSM is a natural step in the evolution of minimally invasive breast surgery that has the potential to mitigate the challenges associated with traditional NSM. In NSM, the upper pole of the breast is the most difficult to access through a submammary incision. In R-NSM, due to the enlarged optic space created as the dissection progresses through the sinus, dissection actually becomes easier the farther away from the access incision.

As the use of robotic nipple-sparing mastectomy continues to rise, improved understanding of the surgical, oncologic, and quality of life outcomes is imperative for appropriate patient selection as well as to better understand indications, limits, advantages, and dangers.

Toesca et al. (16), in a phase III, open-label, single-center, randomized controlled trial involving 80 women with breast cancer (69) or with BRCA mutation (11), compared the outcomes of robotic and open nipple-sparing mastectomy. They concluded that complications were similar among groups supporting the safety of the robotic technique. Quality of life was maintained after robotic mastectomy while it decreased significantly after open surgery. Early follow-up does not confirm the absence of premature local failure.

Yuan et al. (17–19) explored the clinical application of mastectomy with single incision followed by immediate laparoscopic-assisted breast reconstruction with latissimus dorsi muscle flap. Fifteen women with primary early breast cancer, three women with breast ductal carcinoma *in situ*, and seven women with severe plasma cell mastitis were treated with partial mastectomy or total mastectomy, sentinel lymph node biopsy, or axillary lymph node dissection through a breast lateral transverse incision. Subsequent breast reconstruction with latissimus dorsi muscle flap was assisted by laparoscopy. They concluded that the surgical approach introduced is minimally invasive with hidden scar and exceptional esthetic results.

## Conclusions

Especially in patients who undergo post mastectomy radiotherapy, the two-stage reconstruction with video-assisted transaxillary endoscopic approach can find its main indication, using incisions positioned far from the mammary region, offering numerous advantages: reducing the trauma of the tissues, avoiding re-entering the scar of the previous mastectomy and placing a new one at a distance in the axilla, so as to avoid problems and risk of extrusion, improving the visualization with the magnification of the anatomy, allowing a better remodeling of the pocket, and accelerating patient recovery.

The use of the endoscopic approach requires adequate training to operate in very limited fields using a suitable tool which is that of video-assisted surgery. More studies on more patients and with longer follow-ups are needed to compare the effectiveness of this technique with the already standardized open ones.

## Data Availability

The original contributions presented in the study are included in the article/Supplementary Material, further inquiries can be directed to the corresponding author. Raw data are available at the URL: https://zenodo.org/record/7472776#.Y6RNLHbMKUk.
