# Erratum: Superomedial pedicle skin-reducing mastectomy in ptotic and large-sized breasts with two-stage reconstruction through transaxillary video-assisted technique: An effective surgical and anesthetic approach

**DOI:** 10.3389/fsurg.2023.1157569

**Published:** 2023-02-27

**Authors:** Frontiers Production Office

**Affiliations:** Frontiers Media SA, Lausanne, Switzerland

**Keywords:** conservative mastectomies, breast reducing surgery, reduction mammaplasty, endoscopic breast reconstruction, erector spinae block, serratus anterior plane block, opioid-free anesthesia

**An erratum on**
Superomedial pedicle skin-reducing mastectomy in ptotic and large-sized breasts with two-stage reconstruction through transaxillary video-assisted technique: An effective surgical and anesthetic approach By Di Monta G, Marone U, Avino F, Esposito E, Cepparulo V, Morra E, Saponara R, Bifulco F, Cuomo A, Cascella M and Mori S. (2023) Front. Surg. 9:1040602. doi: 10.3389/fsurg.2022.1040602

Due to a production error, two statements are updated.

The funder information for the paper was omitted. The statement should appear below:

Funding Statement

This work was partially supported by the Italian Ministry of Health Ricerca Corrente Funds.

Data Availability Statement

Raw data are available at the URL: https://zenodo.org/record/7472776#.Y6RNLHbMKUk"

The publisher apologizes for this mistake.

The original version of this article has been updated.

